# Advancements in AI-driven drug sensitivity testing research

**DOI:** 10.3389/fcimb.2025.1560569

**Published:** 2025-05-02

**Authors:** Hongxian Liao, Lifen Xie, Nan Zhang, Jinping Lu, Jie Zhang

**Affiliations:** ^1^ Department of Radiology, Zhuhai People’s Hospital, The First School of Clinical Medicine of Guangdong Medical University, Zhuhai, China; ^2^ Department of Radiology, Zhuhai People’s Hospital (Zhuhai Hospital affiliated to Jinan University), Zhuhai, China; ^3^ Department of Oncology, Zhuhai People’s Hospital (Zhuhai Hospital affiliated to Jinan University), Zhuhai, China; ^4^ Department of Clinical Laboratory and Medical Research Center, Zhuhai People’s Hospital(Zhuhai Hospital affiliated to Jinan University), Zhuhai, China

**Keywords:** antimicrobial resistance, antimicrobial susceptibility testing, artificial intelligence, machine learning, whole genome sequencing

## Abstract

Antimicrobial resistance (AMR) constitutes a significant global public health challenge, posing a serious threat to human health. In clinical practice, physicians frequently resort to empirical antibiotic therapy without timely Antimicrobial Susceptibility Testing (AST) results. This practice, however, may induce resistance mutations in pathogens due to genetic pressure, thereby complicating infection control efforts. Consequently, the rapid and accurate acquisition of AST results has become crucial for precision treatment. In recent years, advancements in medical testing technology have led to continuous improvements in AST methodologies. Concurrently, emerging artificial intelligence (AI) technologies, particularly Machine Learning(ML) and Deep Learning(DL), have introduced novel auxiliary diagnostic tools for AST. These technologies can extract in-depth information from imaging and laboratory data, enabling the swift prediction of pathogen antibiotic resistance and providing reliable evidence for the judicious selection of antibiotics. This article provides a comprehensive overview of the advancements in research concerning pathogen AST and resistance detection methodologies, emphasizing the prospective application of artificial intelligence and machine learning in predicting drug sensitivity tests and pathogen resistance. Furthermore, we anticipate future directions in AST prediction aimed at reducing antibiotic misuse, enhancing treatment outcomes for infected patients, and contributing to the resolution of the global AMR crisis.

## 1 Background

AMR represents a significant global public health challenge that poses a severe threat to human health. The World Health Organization (WHO) has issued a warning that by 2050, AMR could become the leading cause of mortality, potentially resulting in over 10 million deaths annually (Stanton et al., 2022). Bacteria that were once treatable with multiple antibiotics have developed extensive resistance. According to a report by the U.S. Centers for Disease Control and Prevention (CDC), 2.8 million Americans are infected with resistant bacteria each year, leading to 35,000 deaths (Eurosurveillance editorial team, 2013). These findings underscore the escalating severity of AMR, which poses significant challenges to patient treatment options and safety. In clinical practice, physicians typically make preliminary diagnoses based on the patient’s symptoms, physical examination results, and laboratory infection indicators. However, the identification of pathogens and their drug susceptibility profiles remains dependent on the culturing of biological samples, a process that generally requires 1 to 3 days. During this period, healthcare professionals frequently depend on empirical antibiotic therapy, which may result in the overuse of antibiotics and consequently intensify antibiotic resistance. Research indicates that enhancing the efficiency of pathogen diagnosis is essential for the prompt treatment of infections, minimizing antibiotic misuse, and mitigating the proliferation of antibiotic resistance (Banerjee and Humphries, 2021). The rapid advancements in AI and medical imaging technologies offer promising prospects for AMR management. DL and ML techniques can predict the drug sensitivity and resistance of pathogens by analyzing clinical imaging and laboratory data, thereby providing precise guidance for antibiotic selection. These technologies hold significant potential in infection monitoring, risk prediction, and the optimization of antibacterial drug usage (Blechman and Wright, 2024).

This article provides a comprehensive overview of various methodologies for the detection of pathogenic bacteria and AST, with a particular focus on the potential applications of AI and ML technologies. The integration of AI technology offers the potential to deliver rapid and accurate decision support for clinicians, thereby enhancing patient outcomes, minimizing the misuse of antibiotics, and addressing the critical issue of antibiotic resistance. Looking ahead, the continued advancement of AI technology is anticipated to yield practical solutions to the challenge of AMR.

## 2 The causes of AMR and its resistance mechanisms

AMR is defined as the capacity of pathogenic microorganisms, including bacteria, viruses, fungi, and parasites, to withstand the effects of therapeutic agents that were previously effective. This phenomenon represents a significant threat to the management of infections and surgical procedures and has been identified as one of the top ten global health threats by the WHO (Zhou et al., 2022). According to the most recent report from the US CDC, drug-resistant infections result in over 35,000 fatalities annually in the United States, with the global mortality figure projected to reach 1.2 million (Eurosurveillance editorial team, 2013; Antimicrobial Resistance Collaborators, 2022).

The unwarranted prescription of antibiotics and their misuse in treating viral infections and other non-bacterial conditions significantly contribute to the accelerated emergence of antibiotic resistance. According to the European Antimicrobial Resistance Surveillance Network (EARS-Net), approximately 25% of antibiotic prescriptions are issued without a legitimate medical justification (European antimicrobial resistance surveillance network (EARS-net), 2017). Furthermore, the advancement of antibacterial drug development has substantially slowed in recent years due to economic and technical challenges. Over the past two decades, fewer than 15 new antibacterial drugs have been approved, a number inadequate to effectively combat the growing problem of resistance (Luepke et al., 2017). Additionally, the lack of rapid and accurate diagnostic tools has led to the widespread empirical use of antibiotics, thereby increasing the selective pressure on resistant bacterial strains (Morrison and Zembower, 2020; Akram et al., 2023).

A comprehensive understanding of antibiotic resistance mechanisms is crucial for effectively addressing this global public health challenge. The historical development of antibiotic resistance mechanisms profoundly illustrates the adaptive strategies employed by microorganisms to withstand environmental stressors. These mechanisms can be categorized into two primary types: intrinsic resistance and acquired resistance. Intrinsic resistance is dictated by the inherent genetic characteristics of bacteria and is prevalent in specific strains. Following the widespread introduction of penicillin in the 1940s, researchers identified that bacterial beta-lactamases could hydrolyze the beta-lactam ring of penicillin, marking a significant milestone in early resistance research (Davies and Davies, 2010). These enzymes inactivate antibiotics by altering their chemical structure, with extended-spectrum beta-lactamases (ESBLs) being particularly prevalent in Escherichia coli and Klebsiella pneumoniae, thus representing a major source of hospital-acquired infections (Morales-León et al., 2020). During the 1960s, an outbreak of methicillin-resistant *Staphylococcus aureus* (MRSA) elucidated the mechanism of drug-resistant target modification. The penicillin-binding protein (PBP2a), encoded by the mecA gene, diminishes the binding efficacy of beta-lactam antibiotics (Davies and Davies, 2010; Ambade et al., 2023).Comprehensive investigations into Gram-negative bacteria have further demonstrated that their outer membrane structure impedes the penetration of antimicrobial agents by down-regulating porin expression. For instance, the intrinsic resistance of Pseudomonas aeruginosa to carbapenems is closely associated with the loss of specific outer membrane proteins (I and S, 2018). In the 1980s, the MexAB-OprM efflux pump system, initially identified in Pseudomonas aeruginosa, was found to confer resistance through the active expulsion of beta-lactams and fluoroquinolones (Davies and Davies, 2010). The efflux pump mechanism is prevalent, as exemplified by Salmonella, which exhibits considerable resistance to polymyxins and aminoglycosides via this mechanism (Shirshikova et al., 2021).

Acquired resistance arises through gene mutation or horizontal gene transfer (HGT), and research in this area has advanced in tandem with developments in molecular biology. The plasmid-mediated horizontal transfer of drug resistance genes was first documented in the 1950s (Davies and Davies, 2010). For instance, the global dissemination of the CTX-M ESBL gene has facilitated the spread of multidrug-resistant bacteria (McNulty et al., 2018; Chis et al., 2022). The clinical outbreak of methicillin-resistant *Staphylococcus aureus* (MRSA) in 1961 underscored the significance of genetic mutations, particularly the mecA gene, which encodes the PBP2a protein, rendering beta-lactam antibiotics ineffective (Davies and Davies, 2010). A comparable mechanism is observed in Acinetobacter baumannii, where gene mutations result in the modification of targets for β-lactams, macrolides, and fluoroquinolones, leading to the failure of these antimicrobial agents (Martinez-Trejo et al., 2022).In the 21st century, plasmids harboring blaNDM-5 genes, such as IncFII and IncX3, have proliferated extensively within E. coli populations. These plasmids frequently co-occur with blaCTX-M, mcr, and other multidrug resistance genes within highly transmissible clones, such as ST167, thereby exacerbating the carbapenem resistance crisis (Blanquart, 2019). Recently, the issue of drug resistance has extended beyond traditional bacterial pathogens. Candida auris, for instance, acquires heat-resistant genes through ERG11 gene mutations, ABC transporter pump efflux mechanisms, such as CDR1, and horizontal gene transfer, leading to cross-resistance against three classes of antifungal drugs and resulting in high mortality rates in hospital-acquired infections (Sabino et al., 2020). Furthermore, carbapenem-resistant Acinetobacter baumannii (CRAB) has developed resistance mechanisms that include efflux pumps, porin deletion, and the integration of blaNDM-1/blaOXA-23 double resistance genes, necessitating the use of colistin as a last-resort treatment (Ijaz et al., 2024). These developments underscore the evolution of antibiotic resistance into a global health security crisis.

AMR represents a substantial threat to global public health by markedly diminishing the efficacy of available treatment options for infections. Furthermore, infections caused by drug-resistant pathogens lead to considerable increases in healthcare expenditures and prolonged hospitalizations. A comprehensive understanding of the mechanisms underlying the development and transmission of AMR is crucial for the formulation of more effective public health policies and the development of innovative antimicrobial strategies (Chis et al., 2022).

## 3 Antibiotic susceptibility testing

AST serves as a critical instrument for evaluating the efficacy of antibiotics against specific bacterial pathogens and is integral to informing anti-infective treatment strategies and optimizing antibiotic utilization. In light of the global health threat posed by AMR, the significance of AST is increasingly pronounced. By precisely determining the drug sensitivity of pathogens, AST aids healthcare professionals in optimizing therapeutic regimens, thereby mitigating antibiotic overuse and curbing the propagation of resistance.The main AST methods, as detailed in **Table 1**.

**Table 1:** Main methods of AST.

Methods	Kirby-Bauer Disk Diffusion	Broth Dilution	E-test (Gradient Diffusion)	Automated AST Systems	Molecular Methods
Principle/Application	Measures zone of inhibition to determine susceptibility	Determines MIC by testing bacterial growth in antibiotic serial dilutions	Uses antibiotic gradient strips to measure MIC directly	Detects bacterial growth via optical/fluorescence signals to calculate MIC	Identifies resistance genes or mutations
Advantages	Simple operation, low cost, suitable for large-scale screening	Provides precise MIC data for personalized therapy	Combines simplicity and precision; ideal for fastidious pathogens	High-throughput, rapid results (4–8 hr), detects complex resistance	Rapid (2–4 hr), predicts resistance in unculturable pathogens or rare mechanisms
limitations	Cannot determine MIC; results influenced by standardization of protocols	Labor-intensive, requires specialized equipment	High reagent costs; limited accessibility in resource-poor regions	Expensive instrumentation and maintenance; requires skilled technicians	Does not assess phenotypic effects; limited to known targets; high costs

**Figure 1:**
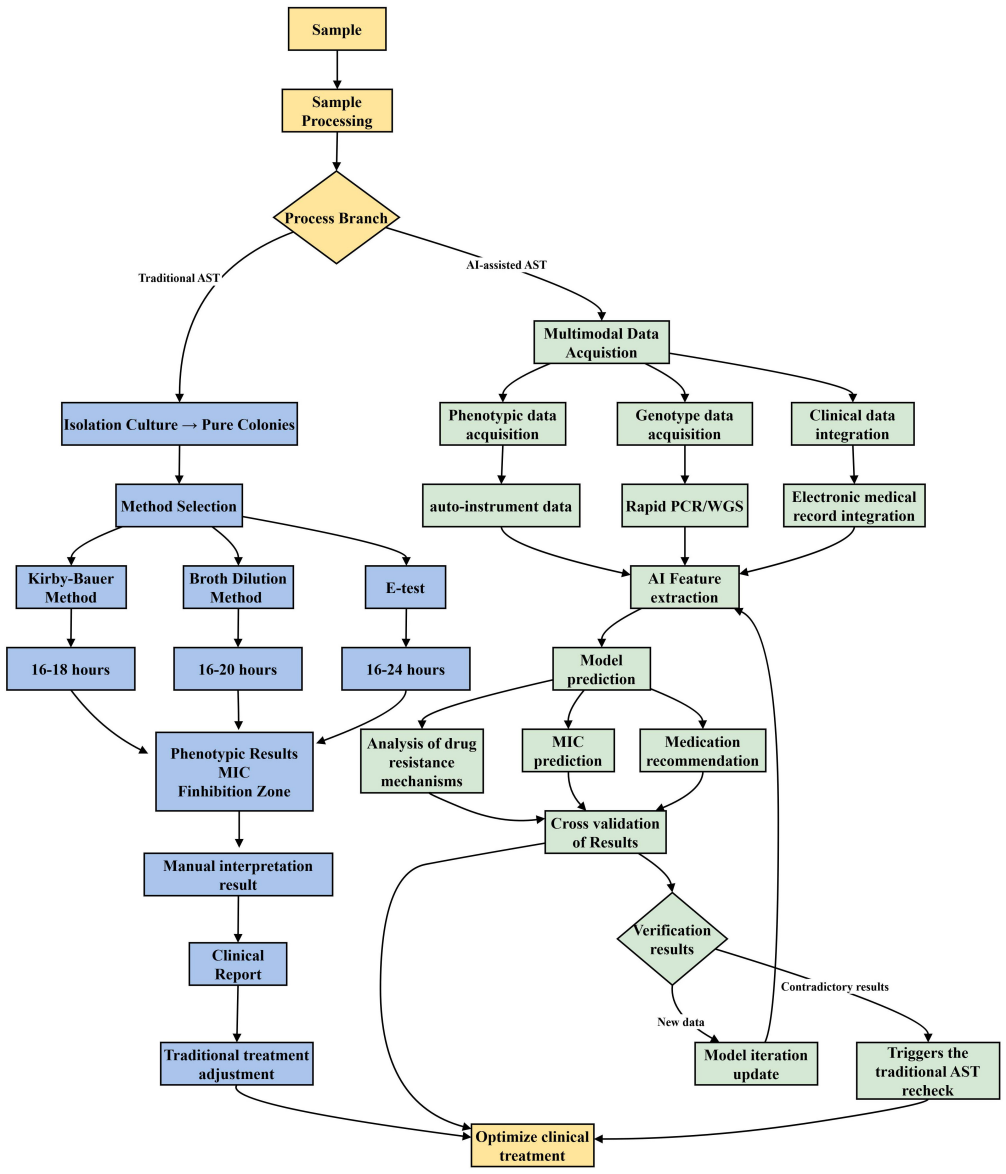
AI-assisted Antimicrobial Susceptibility Trials (AST) enhance clinical decision-making by integrating and analyzing multimodal data. After pretreating clinical samples, phenotypic and genotypic data, along with patient information, are collected. The AI model processes this data to predict drug resistance and suggest personalized treatment plans. If results are contradictory, the model updates using transfer learning. Traditional AST, however, takes 16–24 hours, relying on sample culture, drug sensitivity tests, and manual result interpretation.

Traditional diagnostic methods generally require 24–48 hours to yield results. However, recent advancements in technologies such as real-time microscopy, microfluidic technology, and single-cell analysis have enabled detection within a matter of hours (Behera et al., 2019). These innovative techniques have demonstrated substantial clinical value in studies conducted in Europe and the United States. For example, a study conducted in the United States demonstrated that drug sensitivity could be precisely assessed within a two-hour timeframe using comprehensive electrical monitoring of a microfluidic device (Yang et al., 2020). A comparative flowchart of AI-assisted AST and traditional AST processes is provided in **Figure 1**, highlighting key differences in workflow efficiency and data integration. To advance AST methodologies, Inglis et al. integrated flow cytometer antimicrobial susceptibility testing (FAST) with supervised machine learning techniques. This amalgamation of AI methods facilitates the production of reliable results within a timeframe of less than 3 hours (Inglis et al., 2020). Concurrently, Lechowicz et al. pioneered a novel approach employing an infrared (IR) spectrometer, which synergizes IR spectroscopy with artificial neural networks, thereby reducing the AST duration from 24 hours to a mere 30 minutes (Lechowicz et al., 2013).The innovation of the AST approach not only enhances the precision of antibiotic administration but also provides significant support for the global management of antimicrobial resistance. Looking ahead, the integration of artificial intelligence, genomics, and rapid detection technologies is anticipated to further augment the speed and accuracy of testing. These advancements are expected to optimize clinical treatment strategies and mitigate antibiotic overuse, thereby addressing the resistance crisis.

## 4 Application of AI in AST and resistance prediction

AI and ML technologies are significantly advancing the domain of AST and resistance detection. The integration of AI enables researchers to efficiently analyze extensive biological datasets, thereby predicting bacterial antibiotic sensitivity and resistance with enhanced speed and accuracy, which in turn supports antimicrobial selection and infectious disease management (Boolchandani et al., 2019). Recent years have witnessed substantial advancements in the application of AI and ML within the AST field. Through the analysis of intricate AST datasets and large-scale genomic information, AI methodologies have not only elucidated novel resistance mechanisms but have also introduced innovative strategies for optimizing antibiotic utilization (Liu et al., 2021). Furthermore, AI models possess the capability to forecast trends in drug resistance, offering a proactive approach to global antibiotic management (Chowdhury et al., 2020).

### 4.1 Construction and optimization of ML prediction model

#### 4.1.1 Drug resistance prediction based on whole genome sequencing

The prediction of drug resistance through WGS has been advanced by employing machine learning algorithms, including random forests, support vector machines, and deep learning models such as convolutional neural networks (CNNs). Researchers have effectively developed a model for predicting bacterial resistance utilizing WGS data (Ji et al., 2021).By examining resistance genes, gene mutations, and other pertinent genomic features, these models can rapidly and accurately determine the bacterial resistance spectrum. This capability significantly enhances detection efficiency and provides a crucial foundation for optimizing clinical treatment strategies (Wiatrak et al., 2024).Recent research has demonstrated the high accuracy of machine learning models utilizing WGS data for predicting antibiotic resistance across various bacterial species (Van Camp et al., 2020).Despite the constraints posed by the limited availability of WGS data for aligning with AST outcomes, researchers have pursued innovative methodologies to address this issue.Lueftinger et al. developed a composite model integrating cross-validation and stacked generalization of genomic distance perception, which significantly enhanced the average sensitivity and specificity compared to individual models (Lueftinger et al., 2021).Furthermore, to tackle the intricate variations in genome sequences, researchers have investigated machine learning models for genome sequence characterization using nucleotide k-mers. By extracting k-mer features and integrating three machine learning algorithms, Wang et al. successfully predicted the minimum inhibitory concentration (MIC) of *Staphylococcus aureus* for 10 antimicrobial agents and accurately identified methicillin-resistant *Staphylococcus aureus* (MRSA) (Wang et al., 2022b). Similarly, Gao et al. employed a machine-learning algorithm to predict the MIC of Acinetobacter baumannii against 13 antibacterial agents based on k-mer features, demonstrating robust generalization capabilities (Gao et al., 2024).

With the reduction in WGS costs and the increasing availability of large genomic databases, the development of nonlinear sequence analysis models has emerged as a central focus in machine learning applications. Humphries et al. evaluated a machine learning model developed by Next Gen Diagnostics, which successfully predicted the phenotypic susceptibility of Escherichia coli to cefepime (Humphries et al., 2023). Building on this work, Liu et al. employed rapid feature selection (FFS) and codon mutation detection (CMD) techniques to identify genetic signatures associated with resistance in Klebsiella pneumoniae from genome-wide single nucleotide polymorphism (SNP) data, achieving an area under the curve (AUC) of 0.95 (Liu et al., 2021).Furthermore, Van Camp et al. utilized the XGBoost algorithm to accurately predict the resistance of various gram-negative bacteria to common antibiotics, achieving an AUC of 0.97 (Van Camp et al., 2020).

In summary, machine learning models utilizing WGS data demonstrate exceptional efficacy in predicting bacterial resistance. These models not only accurately identify resistance but also provide a robust scientific foundation for personalized treatment strategies. Looking ahead, the ongoing optimization of algorithms and the expansion of data resources are anticipated to foster a deeper integration of precision medicine with public health prevention and control measures.

#### 4.1.2 AI-assisted rapid AST methods

Microfluidic chips and functional nanomaterials are increasingly employed to extract bacterial cells directly from raw samples, thereby reducing or eliminating the need for traditional bacterial culture processes (Qin et al., 2021; Hong et al., 2023). Additionally, matrix-assisted laser desorption/ionization (MALDI) time-of-flight (TOF) mass spectrometry (MS) has become a prevalent technique for bacterial identification in clinical laboratories (Rodríguez-Sánchez et al., 2016; To et al., 2019).Utilizing mass spectrometry to acquire detailed molecular information about bacterial cells, researchers have integrated machine learning and deep learning techniques for data mining to develop sophisticated analytical strategies. The incorporation of AI with microfluidic technology facilitates the detection of antibiotic sensitivity within a matter of hours. These methodologies enable the rapid and precise assessment of sensitivity by analyzing bacterial growth patterns and metabolic characteristics at varying antibiotic concentrations in real-time. For instance, Riti’s research integrated deep learning with microfluidic technology, enabling the detection of colistin sensitivity in merely 2 hours, thereby streamlining the testing process and enhancing efficiency (Riti et al., 2024).

#### 4.1.3 Detection of drug resistance based on metabolomics

The integration of metabolomics and AI represents a novel approach for predicting bacterial resistance. Metabolomics offers extensive phenotypic data pertinent to resistance studies by capturing alterations in the metabolic profiles of bacteria subjected to antibiotic stress (Kok et al., 2022). Concurrently, AI methodologies leverage this data for modeling and predictive purposes. Recent studies have indicated that this synergistic approach has achieved considerable advancements in the rapid prediction of drug resistance. For instance, Larsen et al. introduced a machine learning methodology employing support vector machines (SVM) by integrating metabolomic and transportomic modeling, which effectively discerned molecular signatures of both pathogenic and non-pathogenic Pseudomonas (Larsen et al., 2014). This approach also identified potential therapeutic targets for antibiotic-resistant Pseudomonas, thereby establishing a novel scientific framework for precision treatment. This development is particularly crucial in addressing the clinical challenges posed by Pseudomonas infections. Subsequent research has demonstrated that artificial intelligence models incorporating multiple omics data—such as genomics, transcriptomics, metabolomics, and proteomics—offer a more comprehensive perspective on resistance predictions (Fortuin and Soares, 2022).For instance,Zhao et al. conducted a comprehensive multi-omics investigation, integrating genomics, proteomics, and metabolomics, to explore capreomycin (CAP) resistance in Mycobacterium tuberculosis (M.tb) strains. Utilizing MetaboAnalyst in conjunction with liquid chromatography-mass spectrometry (LC-MS)-based metabolomics and labeled proteomics techniques, they identified a novel mechanism of CAP resistance linked to tlyA-deficient and mutated M.tb strains (Zhao et al., 2019a). Their research elucidates the complex interactions between genetic modifications and metabolic profiles that contribute to drug resistance in M.tb strains. Furthermore, it facilitates the identification of novel resistance mechanisms, thereby establishing a foundation for the development of more effective antibiotic management strategies.

In conclusion, the integration of metabolomics with artificial intelligence technology markedly enhances the efficiency and precision of predicting bacterial resistance. Furthermore, the incorporation of multi-omics data broadens the applicability of AI models. These advancements in research offer a robust tool for tackling the challenge of resistance and pave the way for novel approaches in antibiotic management and precision therapy.

### 4.2 Application of deep learning in image recognition

The inception of deep learning algorithms can be traced back to 1986, with their conceptual framework inspired by the functions of the biological brain and neurons, while also integrating the theoretical underpinnings of statistics and mathematics (Zhao et al., 2019b). Within the rapidly advancing domain of artificial intelligence, deep learning, particularly through the application of convolutional neural networks (CNNs), has exhibited substantial potential and extensive applicability in the realms of AST and drug resistance analysis. As a leading deep learning algorithm, CNN has achieved noteworthy success in image analysis related to AST. For example, Gullu et al. employed CNN technology to automatically measure the diameter of antibacterial zones and classify antibacterial spectra, thereby effectively assessing bacterial sensitivity and resistance (Gullu et al., 2024).

In recent years, various automated systems leveraging deep learning have been developed. For example, Gerada (Gerada et al., 2024) employed Antilogic software to facilitate the agar dilution method for determining the minimum inhibitory concentration, achieving a basic consistency rate of 98.9% with manual annotations. Hallstrom integrated smartphone image analysis with agar-based tests, such as CombiANT, thereby creating a robust tool for antibiotic resistance analysis (Hallström et al., 2024). Additionally, Brown (Brown et al., 2020) devised an automated and cost-effective optical system capable of delivering early AST results within 4 to 7 hours, significantly reducing incubation time and eliminating human error, while maintaining compatibility with standard phenotypic detection processes.

Furthermore, Bollapragada (Bollapragada et al., 2024) developed a cost-efficient automated AST intelligent system that incorporates IoT, image processing, and deep learning algorithms to enhance the disk diffusion method, thereby reducing the testing duration to 4–6 hours. Karayiğit et al. (2024) introduced a YOLO hybrid model grounded in convolutional neural networks for regional diameter measurement, target detection, and text recognition, which significantly enhances the accuracy and efficiency of the analysis. Yu (Yu et al., 2025) devised an innovative automatic imaging and reporting system that integrates a text recognition model with tone contrast technology to autonomously perform AST interpretation and result reporting via a LINE chatbot, thereby substantially improving convenience and stability.

An increasing number of technologies are being developed to enhance the speed and accuracy of AST. For example, Duong et al (Duong et al., 2023)utilized transfer learning and fine-tuning techniques to attain an F1 score of 0.91 in fluorescence image instance segmentation, offering a novel approach for the precise analysis of antibiotic resistance in Escherichia coli. Similarly, Pyayt introduced an innovative method that integrates machine learning with microscopy techniques, facilitating the rapid capture of bacterial cells and the assessment of antibiotic susceptibility without the necessity for bacterial culture (Pyayt et al., 2020).Additional groundbreaking applications of deep learning have yielded significant outcomes. For example, Xiong’s Parallel Dual-Branch Network (PAS-Net) demonstrates the capability to accurately predict antibiotic susceptibility by analyzing fluorescence images of Pseudomonas aeruginosa (Xiong et al., 2023).Furthermore, the integration of matrix-assisted laser desorption/ionization time-of-flight mass spectrometry (MALDI-TOF MS) with deep learning techniques facilitates the rapid identification of bacterial morphological characteristics and the detection of morphological alterations, such as thickened or deformed cell walls associated with drug resistance. This approach offers a novel methodology for analyzing drug resistance.

Despite the substantial advancements achieved by deep learning in the realms of antimicrobial susceptibility testing and resistance analysis, several challenges persist. Notably, the heterogeneity and scale limitations of datasets impede the generalization capabilities of models, while the interpretability and clinical applicability of these models require further investigation. Looking ahead, the continued optimization of deep learning techniques and the integration of multimodal data are anticipated to significantly enhance the contribution of this field to precision medicine and the management of antimicrobial resistance.

### 4.3 AI-driven AST: scalable and cost-effective pathways to clinical impact

AI-Driven AST enhances the clinical translation process through technological innovation and resource optimization. Technically, the rapid detection of drug resistance phenotypes utilizing a plasma nanosensor array combined with machine learning enables the identification of antibiotic resistance in 12 ESKAPE pathogens within 20 minutes via bacterial fingerprint analysis, achieving an accuracy rate of 89.74%. This provides an efficient tool for precise clinical anti-infection treatment (Yu et al., 2023). Furthermore, by integrating mass spectrometry data with machine learning algorithms such as gradient boosting trees, random forests, or support vector machines (SVM), high-precision detection of carbapenem-resistant Klebsiella pneumoniae (AUC > 0.85) can be accomplished within a few hours, significantly reducing diagnostic time compared to traditional methods (Wang et al., 2022a).Natural language processing (NLP) technology is capable of analyzing microbiological reports and correlating them with patient clinical history data to enhance treatment decisions. Certain systems have the ability to reduce the traditional culture-dependent processing time from several days to just a few hours (Hattab et al., 2024). The dynamic holographic laser speckle imaging (DhLSI) system, as proposed by Yang et al (Yang et al., 2025), when integrated with machine learning algorithms, can assess bacterial sensitivity to antibiotic treatment within 2–3 hours, demonstrating significant time efficiency. These technological advancements not only substantially improve detection efficiency but also confirm their predictive performance and diagnostic accuracy. A meta-analysis of 80 studies indicated that machine learning models surpassed traditional metrics in antimicrobial management (AMS) scenarios, including the area under the curve (AUC) value [ES: 72.28 (70.42–74.14)], accuracy[ES: 74.97 (73.35–76.58)], sensitivity[ES=76.89(71.90–81.89)], and specificity [ES: 73.77; (67.87–79.67)] (Pennisi et al., 2025a).

In the context of resource optimization, the integration of lightweight models with cost-effective equipment significantly reduces the technical barriers. The AI-assisted mobile medical system developed by Ding et al. (2024) employs paper-based β-lactamase fluorescence probe analysis equipment in conjunction with a smartphone AI cloud platform. This approach substantially decreases the cost per detection and reduces reliance on specialized equipment by utilizing a 20-second rapid response fluorescence probe (B1) and cloud-based intelligent calibration. Similarly, Cunningham et al. (2021) integrated the SHERLOCK detection system with smartphone fluorescence imaging, leveraging CRISPR-Cas13a technology to target and identify pathogen genes. This was followed by the rapid analysis of malaria resistance genes using a lightweight classification algorithm, which demonstrated high sensitivity and specificity in resource-constrained settings.The offline artificial intelligence application developed by Pascucci et al. (2021) employs a machine learning model to automatically analyze the bacterial inhibition zone diameters obtained through the Kirby-Bauer disk diffusion method. This application demonstrates a consistency rate of 90% to 98% with the standard method, thereby effectively minimizing inter-operator variability and offering a portable detection solution suitable for resource-limited settings. In clinical practice, the integration of multidimensional data sources, including electronic health records (EHRs), laboratory data, environmental monitoring information, and genomic data, allows AI to optimize antibiotic management processes. This integration enhances diagnostic speed and personalized treatment, curtails the misuse of broad-spectrum antibiotics, and mitigates the emergence of drug resistance (Pennisi et al., 2025b).In conclusion, AI-Driven AST is redefining the diagnostic and therapeutic framework for infectious diseases by leveraging its advantages of rapid processing, cost-effectiveness, and high precision.

## 5 Challenges and future directions

While AI-AST demonstrates significant potential, its clinical implementation is hindered by several challenges, including data bias, limited generalizability, the complexity of integrating multi-omics data, and regulatory and ethical concerns (Ali et al., 2023). Variability in detection methods across laboratories, such as inconsistent MIC breakpoints, introduces data noise that may compromise model reliability (Mouton et al., 2018; Hicks et al., 2019). Additionally, the model’s inconsistent performance with cross-regional data and its low sensitivity to rare drug resistance phenotypes—substantially lower than that for common variants—underscore its limited generalizability (Argimón et al., 2020; Anahtar et al., 2021). Although multi-omics integrated AI models can elucidate the dynamic regulatory networks underlying drug resistance mechanisms (Selevsek et al., 2020), they face issues such as difficulties in aligning heterogeneous data,computational complexity, and the lack of interpretability in deep learning models (e.g., CNNs). These challenges complicate the identification of the responsible factors in cases of misdiagnosis (Ali et al., 2023).Moreover, there is an urgent need to address the risk of medical data privacy breaches and the uneven geographical distribution. The federated learning framework uses collaborative training models to predict individual drug sensitivity, ensuring precise drug guidance while maintaining privacy (Shahsavari et al., 2024; Artificial Intelligence Techniques Based on Federated Learning in Smar).

To effectively address the dynamic evolution of antibiotic resistance (AMR), it is imperative to establish a multi-dimensional data integration capability alongside a robust privacy security framework. Dang et al. (Anh Tuan et al., 2024) successfully integrated quorum sensing (QS) with machine learning to develop an adaptive system capable of predicting the risk of transmission of unknown drug resistance genes, thereby providing early warnings and facilitating a shift in drug resistance management towards proactive prevention and control. Future research and development should prioritize enhancing the generalization capabilities of models, optimizing strategies for multi-omics integration, and constructing an AI framework that balances efficiency with privacy security. Ultimately, the goal is to achieve the comprehensive application of AI-assisted antimicrobial susceptibility testing in clinical practice through ongoing technological innovation and interdisciplinary collaboration.

## 6 Summarize

AI technology is at the forefront of innovation in AST and resistance research, infusing the field of medical microbiology with unprecedented dynamism. By integrating multi-omics data analyses, including genomics, metabolomics, and proteomics, alongside advanced technologies such as deep learning, AI has markedly enhanced the speed and precision of detection processes. This advancement enables not only the rapid prediction of bacterial resistance spectra but also the precise identification of drug-resistant genes and mutation sites. The capability for multi-dimensional data integration offers a robust tool for investigating the mechanisms underlying bacterial drug resistance and paves the way for novel antimicrobial drug development. Recent studies increasingly affirm the potential of AI in this domain. For instance, predictive models leveraging machine learning algorithms can swiftly evaluate bacterial resistance by analyzing gene sequences, while deep learning-driven image analysis techniques can discern potential drug-resistant phenotypes from microscopic images (Chhibbar and Joshi, 2019; Hussain et al., 2020).

Furthermore, AI demonstrates considerable potential in the identification and development of novel antibiotics. Recent studies have employed generative adversarial networks (GANs) to design new molecular structures, thereby significantly reducing the development timeline for drug candidates (Cesaro et al., 2023). Looking ahead, the ongoing refinement of AI algorithms and the continuous growth of biological data repositories suggest an expanded scope for AI applications in predicting antibiotic sensitivity and facilitating personalized treatment strategies. AI is anticipated to enable comprehensive analyses of individual patients’ microbiomes and infection environments, offering tailored treatment options that enhance clinical outcomes and mitigate antibiotic misuse. Additionally, the integration of AI with automated experimental platforms may advance the standardization and quantification of drug resistance research, offering innovative solutions for precision medicine and public health.
